# Limits of pre-endoscopic scoring systems in geriatric patients with upper gastrointestinal bleeding

**DOI:** 10.1038/s41598-024-70577-2

**Published:** 2024-08-30

**Authors:** Giuseppe Di Gioia, Moris Sangineto, Annalisa Paglia, Maria Giulia Cornacchia, Fernando Parente, Gaetano Serviddio, Antonino Davide Romano, Rosanna Villani

**Affiliations:** 1 Liver Unit, Department of Medical and Surgical Sciences, University of Foggia, Foggia, Italy; 2 Internal Medicine Unit, Ospedale Vito Fazzi, Lecce, Italy

**Keywords:** Gastrointestinal bleeding, Geriatric population, UGIB risk scores, Gastroenterology, Predictive markers

## Abstract

Upper gastrointestinal bleeding (UGIB) is a common cause of hospital admission worldwide and several risk scores have been developed to predict clinically relevant outcomes. Despite the geriatric population being a high-risk group, age is often overlooked in the assessment of many risk scores. In this study we aimed to compare the predictive accuracy of six pre-endoscopic risk scoring systems in a geriatric population hospitalised with UGIB. We conducted a multi-center cross-sectional study and recruited 136 patients, 67 of these were 65–81.9 years old (“< 82 years”), 69 were 82–100 years old (“≥ 82 years”). We performed six pre-endoscopic risk scores very commonly used in clinical practice (i.e. Glasgow-Blatchford Bleeding and its modified version, T-score, MAP(ASH), Canada–United Kingdom–Adelaide, AIMS65) in both age cohorts and compared their accuracy in relevant outcomes predictions: 30-days mortality since hospitalization, a composite outcome (need of red blood transfusions, endoscopic treatment, rebleeding) and length of hospital stay. T-score showed a significantly worse performance in mortality prediction in the “≥ 82 years” group (AUROC 0.53, 95% CI 0.27–0.75) compared to “< 82 years” group (AUROC 0.88, 95% CI 0.77–0.99). In the composite outcome prediction, except for T-score, younger participants had higher sensitivities than those in the “≥ 82 years” group. All risk scores showed low performances in the prediction of length of stay (AUROCs ≤ 0.70), and, except for CANUKA score, there was a significant difference in terms of accuracy among age cohorts. Most used UGIB risk scores have a low accuracy in the prediction of clinically relevant outcomes in the geriatric population; hence novel scores should account for age or advanced age in their assessment.

## Introduction

Upper gastrointestinal bleeding (UGIB) is a potentially life-threatening condition defined as bleeding originating from a source proximal to the ligament of Treitz. UGIBs represent a leading cause of hospital admission worldwide with an estimated incidence of 50–100/100,000 individuals per year^1,2^ and mortality rates ranging from 5 to 10%^3^.

Despite the recent advancements in the management of UGIB emergency conditions, the geriatric population remains a high-risk category. Approximately 35–45% of patients referring to the hospital for UGIB are over 60 years of age^4,5^, and estimates are expected to rise due to the aging population in the future. Advanced age critically influences morbidity and mortality rates associated with UGIB^6–8^ and the incidence of complicated gastrointestinal events^5,9^.

Common symptoms of serious UGIB include hematemesis and/or melena, syncope, or hemodynamic failure. In geriatric patients minor clinical symptoms such as dyspepsia, heartburn or abdominal pain, tend to be rare compared to younger individuals, causing a delay in the diagnostic-therapeutic process^10,11^. Furthermore, beyond the high prevalence of risk factors (e.g., use of antiplatelets, anticoagulants, nonsteroidal anti-inflammatory drugs (NSAIDs) or corticosteroids)^8,12^ the presence of multiple comorbidities in geriatric people often provokes severe gastrointestinal adverse events and clinical deterioration after UGIB^8,13^.

In order to predict relevant outcomes including mortality, severity of GI bleeding, rebleeding and length of hospital stay, several risk scores have been proposed mostly taking into account demographic, biochemical, clinical (pre-endoscopic risk scores) and endoscopic data (post-endoscopic risk scores). Prognostic scores reduce healthcare costs without impacting on patient outcomes^14^, by stratifying UGIB in "low-risk", addressed to outpatient management, and “high-risk” patients, requiring hospitalization, urgent endoscopy or intensive care.

However, several prognostic scores do not consider age or advanced age in their assessment. In particular, among the risk scores most frequently used in clinical practice, the Glasgow-Blatchford Bleeding score (GBS)^15^ and its modified version (mGBS)^16^, the T-score^17^ and the MAP(ASH) score^18^ do not consider age as a prognostic factor whereas the Canada–United Kingdom–Adelaide (CANUKA)^19^ and the AIMS65^20^ attribute a score to subjects aged ≥ 65 years although without further differentiation by age group.

In this study we aimed to investigate the validity of those pre-endoscopic risk scores, not considering age or advanced age as a prognostic variable, in the prediction of different outcomes in geriatric subjects hospitalized with UGIB: 30 days mortality since hospitalization, a clinically relevant outcome (required red blood transfusions, endoscopic treatment or rebleeding) and length of hospital stay, in the geriatric population.

## Methods

### Study population and data collection

From November 2021 to May 2022, we conducted a prospective, multi-center and cross-sectional study on the performance of gastrointestinal bleeding risk scores in predicting clinically relevant outcomes in a geriatric population.

Given the aim of this study, patients aged ≥ 65 years with suspected UGIB were recruited whereas severe hematological disorders, inflammatory bowel disease, simultaneous bleeding from a non-gastrointestinal source and lack of data required for calculation of risk scores were considered exclusion criteria. The present study included 136 participants and on the basis of the median age of the study population (82 years old), the total sample was divided into two categories: “< 82 years” (65–81.9 years old) and “≥ 82 years” (82–100 years old).

All individuals visited the Emergency Department of Policlinico Riuniti, Foggia, Italy or the Emergency Department of Hospital “Vito Fazzi”, Lecce, Italy exhibiting symptoms of suspected UGIB including hematemesis and/or melena. Following clinical stabilization patients were hospitalized at the Liver Unit, University Hospital of Foggia or Internal Medicine Unit, Hospital “Vito Fazzi”, Lecce. Medical support was provided to each patient, and clinical signs and laboratory tests were performed at the time of admission. In each case, esophagogastroduodenoscopy (EGDS) was performed within 72 h of admission to the Emergency Department in light of the need for clinical stabilization and the overcrowding of the hospital facilities. All participants who required hemostasis successfully completed the procedure.

The following data were gathered: age, sex, exhibited symptoms at hospital admission (hematemesis, melena or syncope), vital signs (systolic blood pressure, heart rate), mental status, medical history and medication usage. Further, laboratory test findings (haemoglobin, albumin level, blood urea nitrogen, PT, and INR), endoscopic findings, the need for packed red blood cells transfusion, endoscopic management of bleeding and the need for repeated endoscopies due to rebleeding during hospitalization were also collected.

The study was approved by the Ethics Committee of the Teaching Hospital “Policlinico Riuniti” of Foggia and was conducted according to the ethical standards of our institutional research committee and the 1964 Declaration of Helsinki and its later amendments. All participants provided written informed consent to the study.

### Gastrointestinal bleeding risk scores

For this study, the following gastrointestinal bleeding risk scores were considered: Glasgow-Blatchford Bleeding (GBS) and its modified version (mGBS), T-score, MAP(ASH), Canada–United Kingdom–Adelaide (CANUKA), AIMS65. The variables of each scoring system are reported in Table S1. For each risk score, higher values imply higher risks, except for T-scores, for which the mutual score was taken into account.

Due to age variable categorization that prevents comparison with the considered scores, other gastrointestinal bleeding risk scores, such as Clinical Rockall Score, were excluded from this investigation based on the intrinsic methodological limit.

### Assessment of outcomes

Three different outcomes were explored in the present study: mortality, defined as death occurring within 30 days since hospital admission, a composite outcome (required red blood transfusions, endoscopic treatment and rebleeding) and in-hospital length of stay.

Blood transfusion was required in case of haemoglobin < 8 g/dl or hemodynamic instability following medical evaluation.

Endoscopic treatment included hemostatic electrocoagulation, application of endoscopic clips, injection of epinephrine, transarterial embolization or argon plasma coagulation.

Rebleeding was considered in case of bleeding from previously detected or treated lesions during EGDS.

The median of days of hospitalization (11 days) was considered as a threshold to distinguish short (< 11 days) from long (≥ 11 days) hospitalization.

### Statistical analysis

Baseline characteristics of the study participants by age categories (“< 82 years” and “≥ 82 years”) were assessed using chi-square (χ^2^) tests for categorical variables and t-test and Wilcoxon rank sum test for continuous variables. Categorical variables were presented as frequencies or percentages and continuous variables as mean ± SD.

First, the receiver-operating curve (ROC), the area under ROC curve (AUROC), sensitivity, specificity, positive predictive value (PPV), negative predictive value (NPV), odds ratio (OR) and corresponding 95% confidence intervals were calculated to assess the prognostic value of each scoring system for mortality, composite outcome and in-hospital length of stay. ROC curves plot true positive rates (sensitivity) as a function of false positive rates (100-specificity) for different cut-off points and each point on the curve corresponds to a specific decision threshold in terms of sensitivity/specificity.

AUROCs by age categories of each risk score were calculated and tested for equality with the DeLong method^21^. Then, in order to identify the relationship between the explored outcome and risk score in each age category, pairwise comparisons were performed. We examined the relationship between outcome events and each score separately, using logistic regression analysis.

For each scoring system and outcome, the cut-off value as a function of each age category was determined by the maximum product of sensitivity and specificity according to Liu’s method^22^. Then, sensitivity and specificity and their 95% exact binomial confidence intervals were calculated by age categories and compared across different outcomes and risk scores.

Finally, tests of the equality of AUROCs of every risk score by age group were performed.

Values were considered significant if p-value < 0.05 (95% confidence interval). All statistical analyses were performed using Stata SE 15.0 (StataCorp, College Station, TX).

## Results

### Baseline characteristics of the study population

Baseline characteristics of participants by age category are summarized in Table 1. Out of the 136 participants, 67 (49.26%) were in the “< 82 years” group (mean age 74.01 ± 5.44) and 69 (50.74%) were in the “≥ 82 years” group (mean age 88.1 ± 4.26).

**Table 1:** Baseline characteristics of the study population by age categories (n = 136).

	< 82 years (n = 67)	≥ 82 years (n = 69)	*p-*value
Age, years	74.01 ± 5.44	88.10 ± 4.26	< 0.001
Sex (female)	30 (44.78)	42 (60.87)	0.060
Alcohol consumption (current drinkers)	2 (2.99)	1 (1.45)	0.542
Systolic blood pressure	119.41 ± 24.56	116.23 ± 21.29	0.424
Heart rate	89.47 ± 18.48	88.06 ± 17.78	0.652
Haemoglobin	7.71 ± 2.50	7.32 ± 2.36	0.357
Blood urea	71.80 ± 56.85	128.88 ± 201.03	0.026
Albumin	4.78 ± 2.12	3.86 ± 2.36	0.018
INR	1.61 ± 1.73	1.66 ± 1.81	0.854
Heart disease	43 (64.18)	46 (66.67)	0.760
Atrial fibrillation	20 (29.85)	30 (43.48)	0.099
Type 2 diabetes	20 (29.85)	17 (24.64)	0.495
Tumor (any)	11 (16.42)	11 (15.94)	0.940
Liver cirrhosis	5 (7.46)	4 (5.80)	0.696
PPI	37 (55.22)	36 (52.17)	0.721
Corticosteroids	4 (5.97)	3 (4.35)	0.669
Anticoagulants	22 (32.84)	28 (40.58)	0.349
Antiplatelets	27 (40.30)	30 (43.48)	0.707
EGDS findings			
Gastric ulcer	13 (19.40)	16 (23.19)	0.590
Duodenal ulcer	13 (19.40)	17 (24.64)	0.462
Gastritis	15 (22.39)	14 (20.29)	0.765
Tumor	10 (14.93)	6 (8.70)	0.260
Polyps	3 (4.48)	0 (0.00)	0.075
Angiodysplasia	3 (4.48)	0 (0.00)	0.075
GAVE	0 (0.00)	1 (1.45)	0.323
Esophageal varices	4 (5.97)	2 (2.90)	0.383
EGDS timing			0.258
≤ 24 h	30 (44.78)	28 (40.58)	0.621
24–48 h	36 (53.73)	36 (52.17)	0.856
48–72 h	1 (1.49)	5 (7.25)	0.102
UGIB related symptoms			0.940
Melena	46 (68.66)	48 (69.57)	0.909
Hematemesis	14 (20.90)	15 (21.74)	0.904
Melena & hematemesis	7 (10.45)	6 (8.70)	0.728
Deaths	2 (2.99)	8 (11.59)	0.054
Required RBC Transfusion	52 (77.61)	52 (75.36)	0.757
Haemostatic procedures	21 (31.34)	15 (21.74)	0.204
Rebleeding	3 (4.48)	1 (1.45)	0.296
Days of hospitalization	13.29 ± 8.01	14.52 ± 11.44	0.470
GBS score	12.79 ± 2.89	13.79 ± 2.86	0.043
mGBS score	10.70 ± 2.61	11.66 ± 2.61	0.033
MAP (ASH) score	3.46 ± 1.29	4.08 ± 1.41	0.008
CANUKA score	7.64 ± 1.88	8.04 ± 1.99	0.230
T-score	8.38 ± 1.66	8.14 ± 1.45	0.364
AIMS65 score	1.53 ± 0.63	1.87 ± 0.74	< 0.001

Data are presented as means ± standard deviations or number (%).

*INR* international normalized ratio, *PPI* proton pump inhibitors, *EGDS* Esophagogastroduodenoscopy, *RBC* red blood cells, *GBS* Glasgow-Blatchford Bleeding, *mGBS* modified Glasgow-Blatchford Bleeding, *CANUKA* Canada–United Kingdom–Adelaide, *GAVE* Gastric antral vascular ectasia.

Participants in the older group had higher blood urea and lower albumin levels. Moreover, individuals in the older group presented higher scores of GBS, mGBS, CANUKA, MAP(ASH) and T-scores. No differences were found among the groups in terms of antiplatelets, anticoagulants and corticosteroids usage, EGDS findings or UGIB-related symptoms exhibited at the time of admission.

### 30-days mortality since hospitalization

**Fig. 1:**
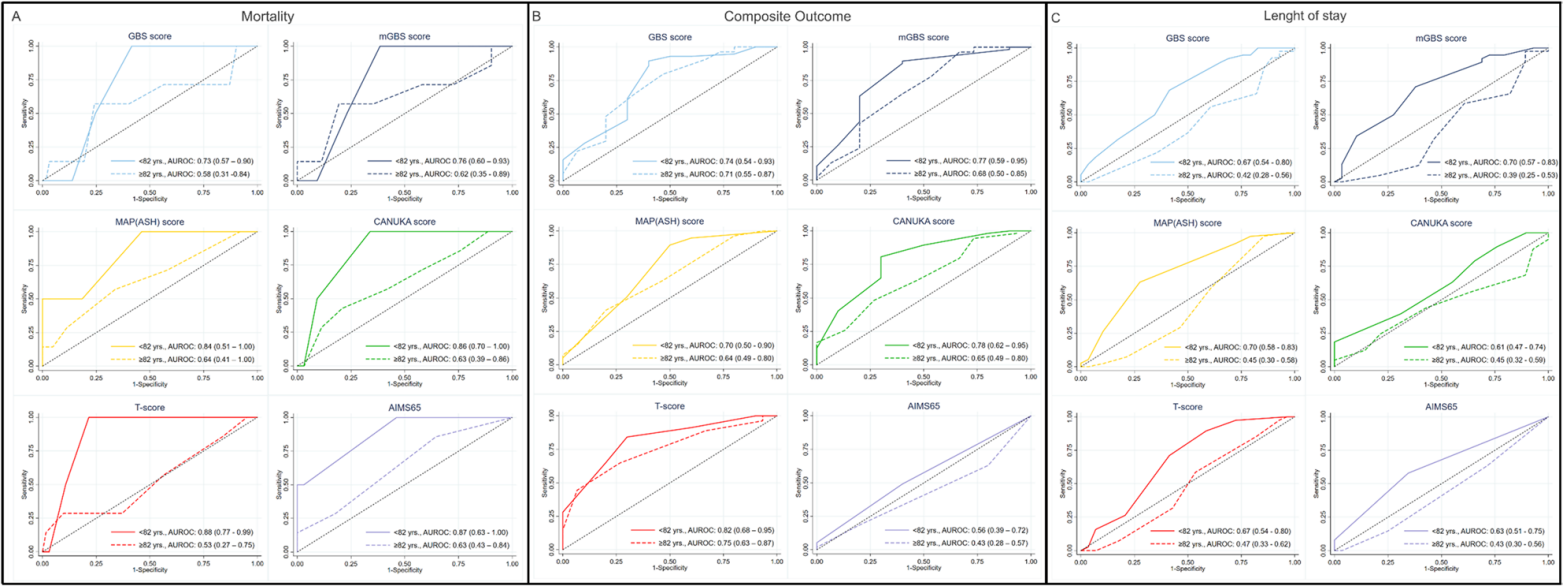
ROC curves of prediction of (**A**) mortality, (**B**) composite outcome and (**C**) length of stay among age groups. The dotted line corresponds to the reference line. “< 82 years.” (65–81.9 years old); “≥ 82 years.” (82–100 years old). *GBS* Glasgow-Blatchford Bleeding, *mGBS* modified Glasgow-Blatchford Bleeding, *CANUKA* Canada–United Kingdom–Adelaide; *yrs.* years.

No scoring system has been shown to be superior in predicting mortality in the overall sample (Table S2). Despite “≥ 82 years” participants showed smaller AUROCs than “< 82 years” participants (Fig. 1A), no significant differences were observed across age groups in predicting mortality among risk scores, except for T-score, where the “< 82 years” group (AUROC 0.88, 95% CI 0.77–0.99) showed a larger AUROC than “≥ 82 years” group (AUROC 0.53, 95% CI 0.27–0.75) (Table 2, Fig. 1A).

**Table 2:** Comparison of ROC curves, sensitivities, and specificities among age categories.

	Mortality				Composite outcome				Length of stay			
	Cut-off	AUROC	Sensitivity (%, 95% CI)	Specificity (%, 95% CI)	Cut-off	AUROC	Sensitivity (%, 95% CI)	Specificity (%, 95% CI)	Cut-off	AUROC	Sensitivity (%, 95% CI)	Specificity (%, 95% CI)
GBS												
< 82 years	13	0.73 (0.57–0.90)	100.00 (15.81–100.00)	58.46 (45.56–70.56)	10	0.74 (0.54–0.93)	**89.5 (78.48**–**96.04)**	60.0 (26.24 -87.84)	12	**0.67 (0.54**–**0.80)**	68.42 (51.35–82.50)	58.62 (38.94–76.48)
≥ 82 years	15	0.58 (0.31–0.84)	57.14 (18.41–90.10)	75.81 (63.26–85.78)	13	0.71 (0.55–0.87)	**64.81 (50.62**–**77.32)**	66.67 (38.38–88.18)	13	**0.42 (0.28**–**0.56)**	56.10 (39.75–71.53)	39.29 (21.50–59.42)
mGBS												
< 82 years	11	0.76 (0.60–0.93)	100.00 (15.81–100.00)	61.54 (48.64–73.35)	8	0.77 (0.59–0.95)	**89.47 (78.48**–**96.04)**	60.00 (26.24–87.84)	10	**0.70 (0.57**–**0.83)**	71.05 (54.10–84.58)	62.07 (42.26–79.31)
≥ 82 years	13	0.62 (0.35–0.89)	57.14 (18.41–90.10)	80.65 (68.63–89.58)	11	0.68 (0.50–0.85)	**64.81 (50.62**–**77.32)**	60.00 (32.29–83.66)	11	**0.39 (0.25**–**0.53)**	58.54 (42.11–73.68)	39.29 (21.50–59.42)
MAP (ASH)												
< 82 years	3	0.84 (0.51–1.00)	100.00 (15.81–100.00)	53.85 (41.03–66.30)	2	0.70 (0.50–0.90)	**89.47 (78.48**–**96.04)**	50.0 (18.71–81.29)	3	**0.70 (0.58**–**0.83)**	63.16 (45.99–78.19)	72.41 (52.76–87.27)
≥ 82 years	4	0.64 (0.41–0.88)	57.14 (18.41–90.10)	66.13 (52.99–77.67)	3	0.64 (0.49–0.80)	**62.96 (48.74**–**75.71)**	53.33 (26.59–78.73)	3	**0.45 (0.30**–**0.58)**	58.54 (42.11–73.68)	39.29 (21.50–59.42)
CANUKA												
< 82 years	8	0.86 (0.70–1.00)	100.00 (15.81–100.00)	66.15 (53.35–77.43)	6	0.78 (0.62–0.95)	**80.70 (68.09**–**89.95)**	70.00 (34.75–93.33)	7	0.61 (0.47–0.74)	63.16 (45.99–78.19)	44.83 (26.45–64.31)
≥ 82 years	8	0.63 (0.39–0.86)	42.86 (9.90–81.59)	79.03 (66.82–88.34)	8	0.65 (0.49–0.80)	**48.15 (34.34**–**62.16)**	73.33 (44.90–92.21)	8	0.45 (0.32–0.59)	43.90 (28.47–60.25)	57.14 (37.18–75.54)
T-score												
< 82 years	8	**0.88 (0.77**–**0.99)**	100.00 (15.81–100.00)	78.46 (66.51–87.69)	10	0.82 (0.68–0.95)	84.21 (72.13–92.52)	70.00 (34.75–93.33)	9	**0.67 (0.54**–**0.80)**	71.05 (54.10–84.58)	58.62 (38.94–76.48)
≥ 82 years	7	**0.53 (0.27**–**0.75)**	28.57 (3.67–70.96)	90.32 (80.12–96.37)	9	0.75 (0.63–0.87)	64.81 (50.62–77.32)	73.33 (44.90–92.21)	9	**0.47 (0.33**–**0.62)**	58.54 (42.11–73.68)	46.43 (27.51–66.13)
AIMS65												
< 82 years	1	0.87 (0.63–1.00)	100.00 (15.81–100.00)	53.85 (41.03–66.30)	1	0.56 (0.39–0.72)	**49.12 (35.63**–**62.71)**	60.00 (26.24–87.84)	1	**0.63 (0.51**–**0.75)**	57.89 (40.82–73.69)	65.52 (45.67–82.06)
≥ 82 years	1	0.63 (0.43–0.84)	85.71 (42.13–99.64)	35.48 (23.74–48.66)	2	0.43 (0.28–0.57)	**18.52 (9.25**–**31.43)**	80.00 (51.91–95.67)	1	**0.43 (0.30**–**0.56)**	63.41 (46.94–77.88)	28.57 (13.22–48.67)

Bold characters: p-value < 0.05.

*AUROC* area under ROC curve, *GBS* Glasgow-Blatchford Bleeding, *mGBS* modified Glasgow-Blatchford Bleeding, *CANUKA* Canada–United Kingdom–Adelaide; *CI* confidence intervals.

In the pairwise comparison of the risk scores among “< 82 years” individuals, CANUKA score showed a larger AUROC compared to GBS score (p-value 0.006) and mGBS score (p-value 0.026). Similarly, in the same group, AUROC of T-score was superior compared to AUROCs of GBS score (p-value 0.007) and mGBS score (p-value 0.027) (Tables S3, S4).

**Fig. 2:**
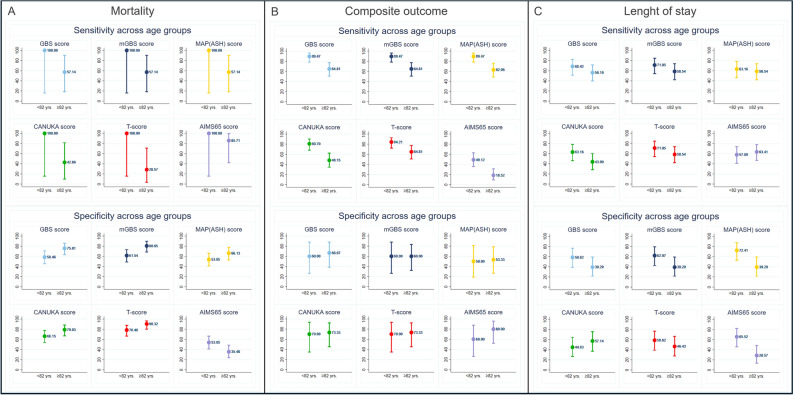
Sensibility and specificity of risk scores predicting (**A**) mortality, (**B**) composite outcome and (**C**) length of stay among age groups. “< 82 yrs.” (65–81.9 years old); “≥ 82 yrs.” (82–100 years old). *GBS* Glasgow-Blatchford Bleeding, *mGBS* modified Glasgow-Blatchford Bleeding, *CANUKA* Canada–United Kingdom–Adelaide.

Moreover, in the mortality prediction risk scores had similar sensitivity and specificity across age groups (Table 2, Fig. 2A).

**Fig. 3:**
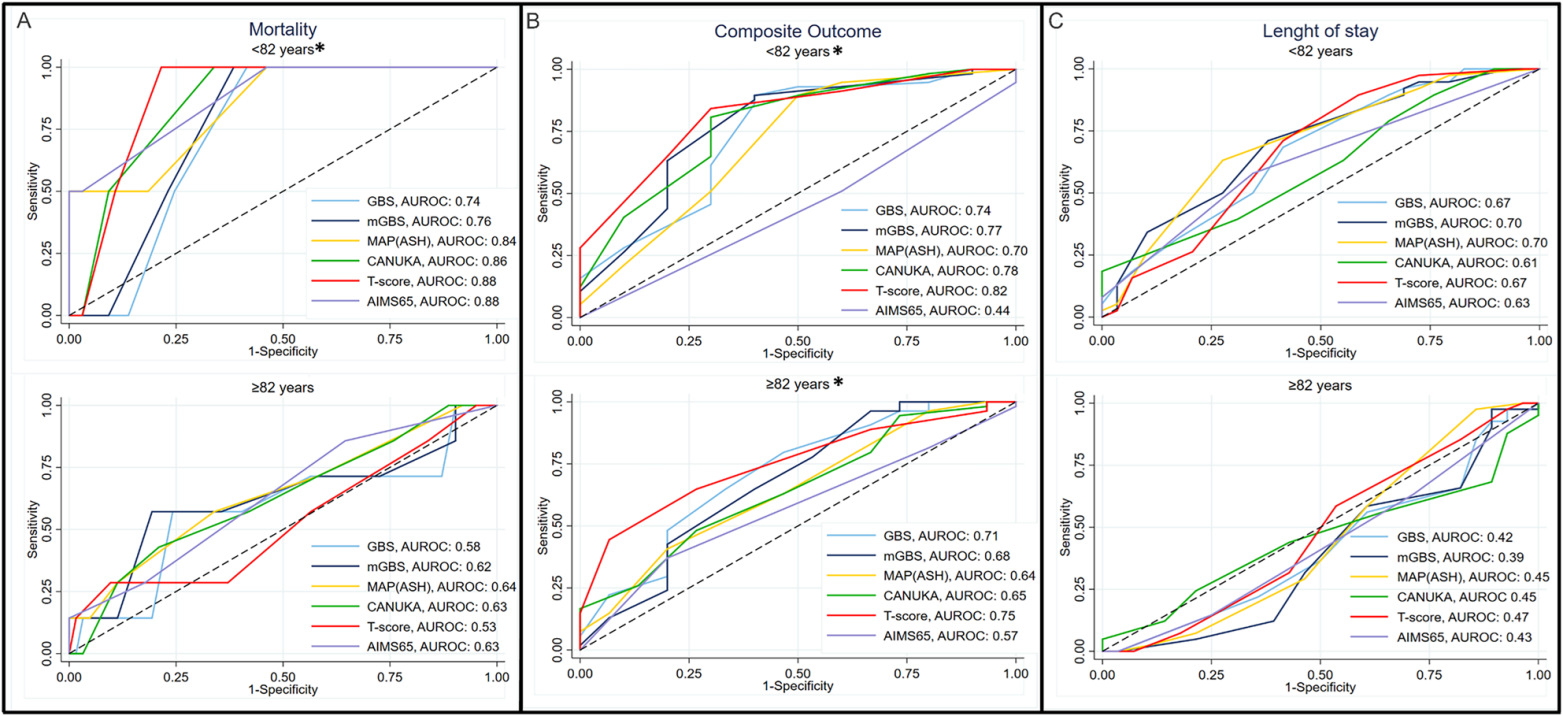
Comparison of risk scores predicting (**A**) mortality, (**B**) composite outcome and (**C**) length of stay among age groups. *Tests of the equality of AUROCs: p-value < 0.05. “< 82 yrs.” (65–81.9 years old); “≥ 82 yrs.” (82–100 years old). *AUROC* area under ROC curve, *GBS* Glasgow-Blatchford Bleeding, *mGBS* modified Glasgow-Blatchford Bleeding, *CANUKA* Canada–United Kingdom–Adelaide.

Comparison of AUROCs revealed no difference in the total sample (p-value 0.145) or in the “≥ 82 years” group (p-value 0.421) but a statistically significant difference in the “< 82 years” group (p-value 0.026) (Fig. 3A).

### Composite outcome

T-score had the largest AUROC compared to the other risk scores in the overall sample (Table S2). No differences across AUROCs (Fig. 1B) and specificities (Fig. 2B) in the prediction of the composite outcome were found among age groups (Table 2). However, all risk scores in the “< 82 years” group had significantly higher sensitivities than those in the “≥ 82 years” group, except for the T-score (Table 2, Fig. 2B).

Pairwise comparison of risk scores among “< 82 years” participants showed inferiority of AIMS65 score compared to mGBS score (p-value 0.016), MAP(ASH) score (p-value 0.049), CANUKA score (p-value 0.011) and T-score (p-value 0.001) (Table S5) whereas no superiority of any risk score was found among “≥ 82 years” participants (Table S6).

Test of equality of AUROCs predicting composite outcome showed a significant difference in the total sample (p-value 0.008), in the “< 82 years” (p-value 0.047) and “≥ 82 years” (p-value 0.048) groups (Fig. 3B).

### Length of stay

All risk scores showed poor and similar performances in predicting length of stay in the overall sample (Table S2) and comparison of AUROCs of each risk score among age groups showed a significant difference in GBS score (p-value 0.010), mGBS score (p-value 0.001), MAP(ASH) score (p-value 0.009), T-score (p-value 0.049) and AIMS65 score (p-value 0.025) with poorer performances in the “≥ 82 years” group but not CANUKA score (p-value 0.113) (Table 2, Fig. 1C).

However, no differences were found in terms of sensitivities and specificities (Table 2—Fig. 2C) or superiority of the risk scores in the pairwise comparison among age groups (Tables S7, S8).

Finally, there was no superiority when comparing AUROCs of risk scores, both in the total sample (p-value 0.796) and among age groups, nor in the “< 82 years” (p-value 0.499) or the “≥ 82 years” (p-value 0.731) groups (Fig. 3C).

## Discussion

Globally, an increasing number of individuals requiring advanced medical support visit Emergency Departments every day, having a significant impact both on patients and on healthcare system in terms of crowding and costs. Patients suffering from suspected UGIB are usually admitted to the Emergency Department with immediate or very urgent priority. However, in order to predict severe outcomes such as mortality, rebleeding, need for urgent endoscopic interventions or RBC transfusions and to detect individuals who require hospitalization, early resuscitation and close monitoring several risk stratification scores have been developed over time. Moreover, to identify those candidates for outpatient management could represent a potential application of UGIB risk scores.

An ideal risk score for UGIB should be easy to calculate, contain accessible variables and predict significant outcomes accurately. To avoid misclassifying “high-risk” patients as “low-risk”, a risk stratification score's sensitivity is important. On the contrary, the specificity of the risk score is less important in terms of patient safety, and more important in terms of resources wastage.

Although endoscopic data would improve UGIB risk scores performance, endoscopy is not continuously available in all medical centres and, in geriatric individuals is often not tolerated or even not applicable^23^.

In contrast, age is a cost-free and easily accessible information which could improve the validity of UGIB risk scores. With the aging population, the higher incidence of UGIB is expected to increase^24^ and the development of a validated tool able to predict clinically relevant outcomes in geriatric patients represent a crucial challenge.

Previous studies highlighted the increasing relative risk associated with advanced age^7^ or implemented existing risk scores with amendments for advanced age, improving the validity of risk stratification^25,26^.

A recently published study compared six pre-endoscopic scoring systems in older (age ≥ 65) and younger (age < 65) cohorts: one of these, the ABC score, a risk score accounting for advanced age (60–74 years; ≥ 75 years), resulted in the best performance in mortality and rebleeding predictions in both age cohorts regardless significant differences between groups whereas MAP(ASH) score was the most accurate for predicting intervention in both groups^27^.

However, most used scoring systems do not consider age or advanced age, generating an important flaw in the evaluation of geriatric patients with UGIB. Here, exploring the validity of most used UGIB scoring systems in the prediction of different clinical outcomes in geriatric individuals, we found that all prognostic scores performed better in the “< 82 years” group (age 65–81.9 years) compared to the “≥ 82 years” group (age 82–100 years) and none of the analysed scores showed a total superiority compared to the others. Moreover, sensitivities were significantly higher in the prediction of the composite outcome in the “< 82 years” group suggesting poor performances in the older population.

Guidelines currently recommend GBS as the most UGIB reliable scoring system^28,29^. In our study, GBS and its modified version mGBS were not superior in the prediction of any of the explored outcomes, neither in age cohorts.

AIMS65 is an easy calculable score system that was initially developed to predict the mean length of stay and mortality^20^; however, here AIMS65 outperformed only in the prediction of mortality in the “< 82 years” group.

MAP(ASH) score accounts for six variables and was initially designed to predict mortality and intervention^18^; here it showed excellent performance in the prediction of mortality in the “< 82 years” group whereas the prediction of composite outcome and length of stay resulted in barely acceptable or very low performances in either age groups.

Similarly, CANUKA score showed acceptable performances among “< 82 years” participants but poor performances in the “≥ 82 years” age group.

Finally, T-score is a simple scoring system including four clinical parameters commonly assessed in the UGIB setting. In the prediction of composite outcome T-score showed superiority compared to the other risk scores in the total sample and both in the “< 82 years” and “≥ 82 years” cohorts. Moreover, in the “≥ 82 years” group T-score was less reliable in predicting mortality than in the “< 82 years” group. This is relevant as Tammaro et al.^30^ demonstrated a similar accuracy to GBS in mortality prediction. On the contrary, T-score was the only score showing a similar sensitivity of composite outcome prediction between “≥ 82 years” and “< 82 years” groups.

### Strengths and limitations

Strengths of this study are the wide and clinically applicable outcomes explored, the prospective and multi-center design and the comparison of pre-endoscopic scoring systems with similar features but different variables. Some limitations should be acknowledged: the event of rehospitalization was not considered, no distinction was made between bleeding due to esophageal varices and other causes, the relatively low number of death events and the ascertainment of the cause of death which could cause mortality to be less accurate. Finally, the results of the analyses cannot be generalized due to the small sample size.

## Conclusions

If on the one hand progresses in modern medicine led to increase life expectancy, from the other multimorbidity and high consumption of drugs such as anticoagulants, antiplatelets or NSAIDs, expose geriatric patients to more adverse outcomes after UGIBs. Several scoring systems are available for UGIB to predict adverse outcomes. This study contributes to the evidence that some of most recommended prognostic scores in clinical practice (i.e., GBS, mGBS, MAP(ASH), CANUKA, T-score and AIMS65) may be biased by not considering age, and especially advanced age, in assessing UGIB severity, resulting in low performance in the prediction of clinically relevant outcomes in the geriatric population. Therefore, we believe that UGIBs scoring systems should be not recommended in advanced age, and further studies are eagerly needed to develop new scores or validate existing ones in old and very old patients.

### Supplementary Information

Supplementary Information.

## Data Availability

All data generated or analysed during this study are included in this published article (and its Supplementary Information file).

## Abbreviations

ASA: American Society of Anaesthesiologists
AUROC: Area under ROC curve
BP: Blood pressure
bpm: Beat per minute
CANUKA: Canada–United Kingdom–Adelaide
EGDS: Esophagogastroduodenoscopy
g/dl: Gram/decilitre
GAVE: Gastric antral vascular ectasia
GBS: Glasgow-Blatchford Bleeding score
HR: Heart rate
INR: International normalized ratio
mGBS: Modified Glasgow-Blatchford Bleeding score
mmHg: Millimetre of mercury
mmol/L: Millimole/litre
NPV: Negative predictive value
NSAID: Nonsteroidal anti-inflammatory drug
OR: Odds ratio
PPI: Proton pump inhibitor
PPV: Positive predictive value
PT: Prothrombin time
RBC: Red blood cells
ROC: Receiver-operating curve
SD: Standard deviation
UGIB: Upper gastrointestinal bleeding
