# Supplementary material for: Limits of pre-endoscopic scoring systems in geriatric patients with upper gastrointestinal bleeding
Source: Sci Rep. 2024 Aug 30;14:20225. doi: 10.1038/s41598-024-70577-2 (PMC11364688; doi:10.1038/s41598-024-70577-2)
Supplement: Supplementary file 1 — Supplementary Information. [file 41598_2024_70577_MOESM1_ESM.docx]

SUPPLEMENTARY

**Table S1.** Variables accounted in the assessment of UGIB risk scores.

| **Glasgow-Blatchford score (maximum score 23) and modified Glasgow-Blatchford score (maximum score 18)** | | | | |
| --- | --- | --- | --- | --- |
|  | | | | |
| Blood urea (mmol/L) | | | Systolic blood pressure (mmHg) | |
| 6.5 – 8 | 2 | | 100 -109 | 1 |
| 8 – 10 | 3 | | 90 – 99 | 2 |
| 10 – 25 | 4 | | <90 | 3 |
| >25 | 6 | | Heart rate (bpm) | |
| Hemoglobin (g/dl), Male | | | ≥100 | 1 |
| 12 - <13 | 1 | | Syncope | 1 |
| 10 - <12 | 3 | | **Additional factors for GBS** | |
| <10 | 6 | | Melena | 1 |
| Hemoglobin (g/dl), Female | | | Liver disease | 2 |
| 10 - <12 | 1 | | Heart failure | 2 |
| <10 | 6 | |  |  |
|  |  | |  |  |
| **MAP (ASH) score (maximum score 9)** | |  | | |
|  | | | | |
| M: altered mental status | 1 | | A: albumin < 2.5 g/dL | 2 |
| A: ASA score > 2 | 1 | | S: Systolic BP < 90 mmHg | 2 |
| P (pulse): HR > 100 | 1 | | H: hemoglobin < 10 g/L | 2 |
|  |  | |  |  |
| **CANUKA Score (maximum score 20)** | | | | |
|  | | | | |
| Age (years) |  | | Hemoglobin (g/dl) |  |
| 50 – 65 | 1 | | 10 – 12 | 1 |
| ≥ 65 | 2 | | 8 – 10 | 2 |
| Heart rate (bpm) |  | | ≤8 | 3 |
| 100 – 125 | 1 | | Blood urea (mmol/L) |  |
| ≥ 125 | 2 | | 5 -10 | 1 |
| Systolic BP (mmHg) |  | | 10 – 15 | 2 |
| 100 – 120 | 1 | | ≥15 | 3 |
| 80 – 100 | 2 | | Melena | 1 |
| <80 | 3 | | Hematemesis | 1 |
| Liver disease | 2 | | Syncope | 1 |
| Malignancy | 2 | |  |  |
|  |  | |  |  |
| **T-score (maximum score 12)**  **[T1 - high-risk <6; T2 – moderate-risk 6-10; T3 – low-risk ≥ 10]** | | | | |
|  |  | |  |  |
| General conditions |  | | Systolic BP (mmHg) |  |
| Poor (≤3 comorbidities/impending to shock) | 1 | | <90 | 1 |
| Intermediate | 2 | | 90-110 | 2 |
| Good (without weakness/orthostatic hypotension and ≤ 1 comorbidity) | 3 | | >110 | 3 |
| Pulse (bpm) |  | | Hemoglobin (g/dl) |  |
| >110 | 1 | | ≤8 | 1 |
| 90-110 | 2 | | 8-10 | 2 |
| <90 | 3 | | >10 | 3 |
|  | | | | |
| **AIMS65 (maximum score 5)** | | | | |
|  | | | | |
| Albumin < 3 (g/dL) | 1 | Systolic BP ≤90 (mmHg) | | 1 |
| INR>1.5 | 1 | Age ≥65 (years) | | 1 |
| Alteration in mental status | 1 |  | |  |

Abbreviations: mmol/L, millimole/litre; g/dl, gram/decilitre; mmHg, millimetre of mercury; bpm, beat per minute; ASA, American Society of Anaesthesiologists; HR, heart rate; BP, blood pressure; INR, international normalized ratio; GBS, Glasgow-Blatchford Bleeding.

**Table S2.** Summary of characteristics of risk scores predicting mortality, composite outcome and length of stay in the overall sample.

|  |  |  |  |  |  |  |  |  |
| --- | --- | --- | --- | --- | --- | --- | --- | --- |
|  | **Cut-off** | **AUROC (95% CI)** | **Sensitivity (%, 95% CI)** | **Specificity (%, 95% CI)** | **PPV (%, 95% CI)** | **NPV (%, 95% CI)** | **OR (95% CI)** | **p-value** |
|  |  |  |  |  |  |  |  |  |
| **Mortality** |  |  |  |  |  |  |  |  |
| GBS | 13 | 0.63 (0.43 – 0.84) | 77.8 (40.0 – 97.2) | 50.4 (41.03 – 59.8) | 10.1 (4.2 -19.8) | 97.0 (89.6 - 99.6) | 3.67 (0.73 - 18.34) | 0.113 |
| mGBS | 11 | 0.67 (0.47 – 0.87) | 77.8 (40.0 – 97.2) | 53.0 (43.5 – 62.3) | 10.3 (4.2 - 20.1) | 97.1 (89.8 - 99.6) | 3.79 (0.76 - 18.93) | 0.105 |
| MAP (ASH) | 4 | 0.71 (0.53 – 0.90) | 55.6 (21.2 – 86.3) | 73.5 (64.5 – 81.2) | 13.2 (4.4 - 28.1) | 95.9 (89.9 - 98.9) | 3.56 (0.90 - 14.04) | 0.070 |
| CANUKA | 8 | 0.69 (0.50 – 0.87) | 66.7 (29.9 – 92.5) | 64.1 (54.7 – 72.8) | 11.1 (4.2 - 22.6) | 96.3 (89.7 - 99.2) | 3.29 (0.79 - 13.77) | 0.103 |
| T-score | 8 | 0.62 (0.41 – 0.83) | 44.4 (13.7 – 78.8) | 69.2 (60.0 – 77.4) | 9.8 (2.7 - 23.1) | 94.7 (88.1 - 98.3) | 1.95 (0.53 - 7.11) | 0.341 |
| AIMS65 | 1 | 0.73 (0.56 – 0.89) | 88.9 (51.8 – 99.7) | 45.3 (36.1 – 54.8) | 10.3 (4.5 - 19.2) | 98.3 (90.8 -100.0) | 6.51 (0.79 – 53.62) | 0.081 |
|  |  |  |  |  |  |  |  |  |
| **Composite outcome** |  |  |  |  |  |  |  |  |
| GBS | 12 | 0.70 (0.58 - 0.83) | 70.3 (60.9 - 78.6) | 60.0 (38.7 - 78.9) | 88.6 (80.1 - 94.4) | 31.3 (18.7 (46.3) | 3.55 (1.47 - 8.56) | **0.006** |
| mGBS | 10 | 0.70 (0.57 – 0.83) | 70.3 (60.9 - 78.6) | 60.0 (38.7 - 78.9) | 88.6 (80.1 - 94.4) | 31.3 (18.7 - 46.3) | 3.55 (1.47 - 8.56) | **0.006** |
| MAP (ASH) | 3 | 0.65 (0.53 – 0.78) | 56.8 (47.0 - 66.1) | 60.0 (38.7 - 78.9) | 86.3 (76.2 - 93.2) | 23.8 (14.0 - 36.2) | 1.97 (0.83 - 4.69) | 0.133 |
| CANUKA | 6 | 0.69 (0.58 – 0.81) | 80.2 (71.5 - 87.1) | 48.0 (27.8 - 68.7) | 87.3 (79.2 - 93.0) | 35.3 (19.7 - 53.5) | 3.73 (1.52 - 9.19) | **0.005** |
| T-score | 9 | 0.77 (0.68 – 0.86) | 64.9 (55.2 - 73.7) | 76.0 (54.9 - 90.6) | 92.3 (84.0 - 97.1) | 32.8 (21.0 - 46.3) | 5.85 (2.21 - 15.39) | **0.001** |
| AIMS65 | 1 | 0.46 (0.35 – 0.57) | 55.9 (46.1 - 65.3) | 36.0 (18.0 - 57.5) | 79.5 (68.8 - 87.8) | 15.5 ( 7.3 - 27.4) | 0.71 (0.30 - 1.72) | 0.458 |
|  |  |  |  |  |  |  |  |  |
| **Length of stay** |  |  |  |  |  |  |  |  |
| GBS | 13 | 0.54 (0.44 - 0.64) | 53.1 (41.5 - 64.4) | 52.6 (38.9 - 66.0) | 60.9 (48.4 - 72.4) | 44.8 (32.6 - 57.4) | 1.26 (0.64 - 2.49) | 0.505 |
| mGBS | 11 | 0.55 (0.45 - 0.65) | 54.4 (42.8 - 65.6) | 56.1 (42.3 - 69.2) | 63.2 (50.7 - 74.6) | 47.1 (34.8 - 59.6) | 1.53 (0.77 - 3.02) | 0.225 |
| MAP (ASH) | 3 | 0.58 (0.48 - 0.68) | 60.7 (49.1 - 71.5) | 56.1 (42.3 - 69.2) | 65.8 (53.7 - 76.5) | 50.8 (37.9 - 63.6) | 1.98 (1.00 - 3.94) | **0.050** |
| CANUKA | 8 | 0.53 (0.43 - 0.63) | 41.7 (30.7 - 53.4) | 63.1 (49.3 - 75.5) | 61.1 (46.9 - 74.1) | 43.9 (33.0 - 55.3) | 1.23 (0.61 - 2.46) | 0.562 |
| T-score | 9 | 0.58 (0.48 - 0.69) | 64.55 (52.9 - 74.9) | 52.63 (38.9 - 66.0) | 65.4 (53.8 - 75.8) | 51.7 (38.2 - 65.0) | 2.02 (1.01 - 4.04) | **0.047** |
| AIMS65 | 1 | 0.53 (0.44 - 0.62) | 60.7 (49.1 - 71.5) | 47.36 (33.9 - 61.0) | 61.5 (49.8 - 72.3) | 46.6 (33.3 - 60.1) | 1.39 (0.70 - 2.76) | 0.345 |
|  |  |  |  |  |  |  |  |  |

Abbreviations: AUROC, area under ROC curve; PPV, positive predictive value; NPV, negative predictive value; OR, odds ratio; GBS, Glasgow-Blatchford Bleeding; mGBS, modified Glasgow-Blatchford Bleeding; CANUKA, Canada-United Kingdom-Adelaide; CI, confidence intervals.

**Table S3. Comparison of risk scores predicting mortality in “<82 yrs.” participants.**

|  | **GBS** | **mGBS** | **MAP(ASH)** | **CANUKA** | **T-score** | **AIMS65** |
| --- | --- | --- | --- | --- | --- | --- |
| **GBS** |  |  |  |  |  |  |
| **mGBS** | 0.312 |  |  |  |  |  |
| **MAP(ASH)** | 0.670 | 0.758 |  |  |  |  |
| **CANUKA** | **0.006** | **0.026** | 0.923 |  |  |  |
| **T-score** | **0.007** | **0.027** | 0.826 | 0.642 |  |  |
| **AIMS65** | 0.484 | 0.578 | 0.378 | 0.939 | 0.964 |  |

**Table S4. Comparison of risk scores predicting mortality “≥82 yrs.” participants.**

|  | **GBS** | **mGBS** | **MAP(ASH)** | **CANUKA** | **T-score** | **AIMS65** |
| --- | --- | --- | --- | --- | --- | --- |
| **GBS** |  |  |  |  |  |  |
| **mGBS** | 0.054 |  |  |  |  |  |
| **MAP(ASH)** | 0.638 | 0.870 |  |  |  |  |
| **CANUKA** | 0.581 | 0.958 | 0.905 |  |  |  |
| **T-score** | 0.653 | 0.446 | 0.373 | 0.355 |  |  |
| **AIMS65** | 0.737 | 0.939 | 0.930 | 0.949 | 0.424 |  |

**Table S5. Comparison of risk scores predicting composite outcome in “<82 yrs.” participants.**

|  | **GBS** | **mGBS** | **MAP(ASH)** | **CANUKA** | **T-score** | **AIMS65** |
| --- | --- | --- | --- | --- | --- | --- |
| **GBS** |  |  |  |  |  |  |
| **mGBS** | 0.452 |  |  |  |  |  |
| **MAP(ASH)** | 0.691 | 0.294 |  |  |  |  |
| **CANUKA** | 0.288 | 0.875 | 0.360 |  |  |  |
| **T-score** | 0.447 | 0.655 | 0.296 | 0.662 |  |  |
| **AIMS65** | 0.086 | **0.016** | **0.049** | **0.011** | **0.001** |  |

**Table S6. Comparison of risk scores predicting composite outcome in “≥82 yrs.” participants.**

|  | **GBS** | **mGBS** | **MAP(ASH)** | **CANUKA** | **T-score** | **AIMS65** |
| --- | --- | --- | --- | --- | --- | --- |
| **GBS** |  |  |  |  |  |  |
| **mGBS** | 0.110 |  |  |  |  |  |
| **MAP(ASH)** | 0.430 | 0.697 |  |  |  |  |
| **CANUKA** | 0.444 | 0.721 | 0.972 |  |  |  |
| **T-score** | 0.659 | 0.430 | 0.117 | 0.158 |  |  |
| **AIMS65** | 0.185 | 0.331 | 0.550 | 0.544 | 0.089 |  |

**Table S7. Comparison of risk scores predicting length of stay in “<82 yrs.” participants.**

|  | **GBS** | **mGBS** | **MAP(ASH)** | **CANUKA** | **T-score** | **AIMS65** |
| --- | --- | --- | --- | --- | --- | --- |
| **GBS** |  |  |  |  |  |  |
| **mGBS** | 0.208 |  |  |  |  |  |
| **MAP(ASH)** | 0.557 | 0.927 |  |  |  |  |
| **CANUKA** | 0.349 | 0.149 | 0.141 |  |  |  |
| **T-score** | 0.963 | 0.640 | 0.628 | 0.346 |  |  |
| **AIMS65** | 0.641 | 0.375 | 0.229 | 0.772 | 0.617 |  |

**Table S8. Comparison of risk scores predicting length of stay in “≥82 yrs.” participants.**

|  | **GBS** | **mGBS** | **MAP(ASH)** | **CANUKA** | **T-score** | **AIMS65** |
| --- | --- | --- | --- | --- | --- | --- |
| **GBS** |  |  |  |  |  |  |
| **mGBS** | 0.351 |  |  |  |  |  |
| **MAP(ASH)** | 0.586 | 0.344 |  |  |  |  |
| **CANUKA** | 0.550 | 0.285 | 0.965 |  |  |  |
| **T-score** | 0.316 | 0.178 | 0.648 | 0.752 |  |  |
| **AIMS65** | 0.889 | 0.659 | 0.756 | 0.753 | 0.555 |  |
